# BETTER HEALTH: Durham -- protocol for a cluster randomized trial of BETTER in community and public health settings

**DOI:** 10.1186/s12889-017-4797-3

**Published:** 2017-09-29

**Authors:** Lawrence Paszat, Rinku Sutradhar, Mary Ann O’Brien, Aisha Lofters, Andrew Pinto, Peter Selby, Nancy Baxter, Peter D. Donnelly, Regina Elliott, Richard H. Glazier, Robert Kyle, Donna Manca, Mary-Anne Pietrusiak, Linda Rabeneck, Nicolette Sopcak, Jill Tinmouth, Becky Wall, Eva Grunfeld

**Affiliations:** 1 Sunnybrook Research Institute, Sunnybrook Health Sciences Centre, University of Toronto, 2075 Bayview Avenue, Toronto, ON M4N3M5 Canada; 2 Institute for Clinical Evaluative Sciences, University of Toronto, St. Michael’s Hospital, 2075 Bayview Avenue, Toronto, ON M4N3M5 Canada; 3 Department of Family and Community Medicine, University of Toronto, 500 University Avenue, Toronto, ON M5G1V7 Canada; 4 University of Toronto, St Michael’s Hospital Family Practice Unit, 30 Bond St, Toronto, ON M5B1W8 Canada; 5 University of Toronto, 410 Sherbourne St, 4th Floor, Toronto, ON M5 Canada; 6 University of Toronto, Centre for Addiction and Mental Health, 175 College Street, Toronto, ON M5T1R8 Canada; 7 University of Toronto, St Michael’s Hospital, 30 Bond Street, Toronto, ON M5B1W8 Canada; 8 Institute of Health Policy, Management, and Evaluation, Dalla Lana School of Public Health, University of Toronto, 155 College Street, Toronto, ON M5T 3M7 Canada; 9 Durham Region Health Department, 605 Rossland Road E, Whitby, ON L1N6A3 Canada; 10 Department of Family Medicine, Grey Nuns Family Medicine Clinic, University of Alberta, 2927 - 66th St NW, Edmonton, AB T6K4C1 Canada; 11 Cancer Care Ontario, 620 University Avenue, Toronto, ON M5 Canada; 12 Department of Family Medicine, University of Alberta, 6 - 10 University Terrace, Edmonton, AB T6G2T4 Canada; 13 University of Toronto, Sunnybrook Health Sciences Centre, 2075 Bayview Avenue, Toronto, ON M4N3M5 Canada

**Keywords:** Chronic disease, Prevention, Screening, Cluster randomized trial, Deprivation

## Abstract

**Background:**

The Building on Existing Tools to Improve Chronic Disease Prevention and Screening (BETTER) cluster randomized trial in primary care settings demonstrated a 30% improvement in adherence to evidence-based Chronic Disease Prevention and Screening (CDPS) activities. CDPS activities included healthy activities, lifestyle modifications, and screening tests. We present a protocol for the adaptation of BETTER to a public health setting, and testing the adaptation in a cluster randomized trial (BETTER HEALTH: Durham) among low income neighbourhoods in Durham Region, Ontario (Canada).

**Methods:**

The BETTER intervention consists of a personalized prevention visit between a participant and a prevention practitioner, which is focused on the participant’s eligible CDPS activities, and uses Brief Action Planning, to empower the participant to set achievable short-term goals. BETTER HEALTH: Durham aims to establish that the BETTER intervention can be adapted and proven effective among 40–64 year old residents of low income areas when provided in the community by public health nurses trained as prevention practitioners. Focus groups and key informant interviews among stakeholders and eligible residents of low income areas will inform the adaptation, along with feedback from the trial’s Community Advisory Committee. We have created a sampling frame of 16 clusters composed of census dissemination areas in the lowest urban quintile of median household income, and will sample 10 clusters to be randomly allocated to immediate intervention or six month wait list control. Accounting for the clustered design effect, the trial will have 80% power to detect an absolute 30% difference in the primary outcome, a composite score of completed eligible CDPS actions six months after enrollment. The prevention practitioner will attempt to link participants without a primary care provider (PCP) to a local PCP. The implementation of BETTER HEALTH: Durham will be evaluated by focus groups and key informant interviews.

**Discussion:**

The effectiveness of BETTER HEALTH: Durham will be tested for delivery in low income neighbourhoods by a public health department. Trial Registration: NCT03052959, registered February 10, 2017.

## Background

Our previous work identified census dissemination areas in Ontario (Canada) within the lowest quintile of median household income. Persons living in these areas seem to face a variety of barriers accessing primary care service as they have low cancer screening rates, are least likely to have a regular primary care provider, and have the lowest average number of primary care visits [1]. In Ontario, primary care providers (PCPs) typically provide screening for cervical, colorectal, and breast cancer, and assessment and screening for cardiovascular and diabetes risk factors. Despite increased funding for Patient Enrollment Models of primary care, and financial incentives to PCPs for cancer screening, access to primary care and participation in cancer screening in Ontario have not improved, particularly not in low imcome areas [1–3]. Two notable strategies have been previously implemented: 1) community health centres, targetting those with multiple morbidities in low income areas, and 2) incentive payments to primary care providers for cancer screening participation by their patients. However neither of these have had an impact on screening rates among low income areas overall [1, 4].

The BETTER cluster randomized trial (BETTER = Building on Existing Tools To Improve Chronic Disease Prevention and Screening) intervention [5] focused on evidence-based Chronic Disease Prevention and Screening (CDPS) activities identified by systematic literature review [6]. A prevention practitioner (a new role undertaken by allied health professionals within a primary care practice) met in a personalized, one-on-one visit with participants to improve their uptake of CDPS activities. This intervention was demonstrated to be effective in a cluster randomized trial among primary care clinics [5].

This paper presents the plans for adaptation of the BETTER intervention for delivery to persons aged 40–64 years living in low income communities by a public health unit, staffed by public health nurses who have experience in addressing social determinants of health, and who have received prevention practitioner training. Public health units in Ontario are focused on the prevention of acute and chronic diseases and have mandates to promote healthy lifestyles and activities as well as chronic disease screening; these units work alongside primary care providers and community health centres, but do not provide primary care services. The adaptation of BETTER will be evaluated qualitatively, and the effectiveness of the adapted intervention will be tested in a cluster randomized trial, among low income areas in Durham Region, Ontario. The rationale for adapting BETTER in the community and public health setting for residents of low income neighbourhoods is that their burden of chronic diseases and mortality is higher [7], participation in chronic disease screening is lower [1], and primary care visits are fewer in such neighbourhoods [1]. The rationale for the cluster randomized design is to account for the variable opportunities for CDPS actions among low income communities.

The BETTER intervention was originally designed with several goals: to integrate the approach to CDPS actions, to optimize participation in evidence-based CDPS actions in primary care practices, and to create a feasible, evidence-based intervention to motivate participants to undertake CDPS actions for which they are eligible but not currently undertaking [5, 6]. The first BETTER cluster randomized trial compared the outcomes of the BETTER prevention practitioner intervention at the individual patient level within allocated clusters of PCPs, to outcomes within clusters of PCPs allocated to a six-month wait list control group.

For each participant in the original BETTER trial, their status for each of 28 CDPS activities was determined at baseline. CDPS included process measures derived from the electronic medical record (EMR) such as the presence or absence of (1) biometrics (body mass index and blood pressure), (2) record of screening for alcohol, smoking, exercise and nutritional status, (3) uptake of glucose and lipid testing, (3) calculated Framingham score, and (4) results cancer screening (cervical, colorectal and breast). Eligibility for other CDPS actions, and their presence or absence, were determined from the information collected by a baseline health survey (e.g. family history, treatments for hypertension, dyslipidemia and diabetes, cancer screening history, nutrition, exercise, smoking, alcohol consumption). Six months after study entry, the status of each participant regarding each CDPS for which the participant was eligible was determined from the EMR and a followup health survey, capturing updated information for all of the above plus any referrals for, or participation in lifestyle-modifying behaviours that occurred during the previous six months.

The BETTER intervention consisted of a one-on-one personalized meeting between a specially trained prevention practitioner and the participant. The prevention practitioner reviewed the status of all CDPS actions with the participant, using the Better Program visual aids [8] and summarizing all CDPS actions for which the individual was eligible. Using principles of Brief Action Planning [9, 10], shared decision making, and health coaching, the prevention practitioner empowered the individual to set a limited number of feasible, self-prioritized short term goals for accomplishing a limited number eligible CDPS actions during the following one or two weeks, summarized on a Goal Sheet [8].

Even though the Brief Action Planning focuses on a limited number of short term goals, the longer term impact of the visit with the prevention practitioner was demonstrated by the large difference seven months later in the percentage of completed CDPS actions between those who received the intervention immediately and those who had been randomized to the wait-list control arm. The main outcome measure, a composite index score, consisted of the ratio (multiplied by 100) of the number of eligible CDPS actions at baseline (denominator) that have been accomplished at follow-up (numerator) [5, 11]. In the adjusted analysis, participants in the wait-list control clusters met 23.1% (95% CI: 19.2% to 27.1%) of target actions, compared to 55.6% (95% CI: 49.0% to 62.1%) met by those in the immediate intervention clusters *p* < 0.001) [5]. This result was replicated in an implementation study (BETTER 2) among primary care practices in communities in Newfoundland; contextual factors, such as the health services environment, availability of resources, attributes of the individual prevention practitioners, and stakeholder engagement were found to have a strong influence on the success of implementation of the BETTER intervention [12–15].

### Training of prevention practitioners

The BETTER intervention is supported by a training program for prevention practitioners, based on the BETTER tools including baseline and followup patient questionnaires [8], and visual aids for explaining CDPS activities and assisting the patient to set goals for accomplishing CDPS activities as prioritized by the participant [8]. After their training, prevention practitioners discuss their experience with the BETTER intervention and Brief Action Planning among themselves, and with investigators or supervisors. Individuals may apply for certification in Brief Action Planning [10], but there is no method for ongoing quantitative assessment of fidelity to the principles of Brief Action Planning.

### Aims of BETTER HEALTH: Durham

The goal of ‘BETTER HEALTH: Durham’ is to improve participation in CDPS actions in low income areas, by adapting the ‘BETTER’ intervention for delivery by the Durham Region Health Department (public health unit), and testing the effectiveness of the adaptation in a cluster randomized trial.

The specific objectives of BETTER HEALTH: Durham are:

(1) To detect a 30% improvement in adherence to evidence-based CDPS actions among individual participants in intervention clusters, 6 months after the BETTER intervention, compared to individual participants in wait-list control clusters, as observed in the first BETTER cluster randomized trial [5]. The 30% increase is measured by the composite BETTER index score and reflects eligible CDPS actions met within six months following enrollment, between the immediate intervention clusters compared to the ‘wait-list’ control clusters (as achieved in the original BETTER cluster randomized trial). All participants will complete an outcome survey six months following the baseline survey; those in ‘immediate intervention’ clusters will have been offered the intervention immediately after completion of the baseline survey whereas those in ‘wait-list’ control clusters will be offered the BETTER intervention after completion of the outcome survey.(2) To adapt and tailor the BETTER intervention to the populations of the eligible low income areas using a community engagement strategy and participatory research methods, including assessing the social determinants of health of participants and linking them to community resources.(3) To evaluate the implementation of the adapted BETTER HEALTH: Durham intervention among participants residing in the eligible low income areas.(4) To conduct integrated knowledge translation with participants residing in eligible low income areas, stakeholders and knowledge users throughout the project and at the end of the study [16].

## Methods/design

### Setting

Census dissemination areas in Durham Region, Ontario, Canada, classified in the lowest quintile of urban median household income, with low cancer screening participation and low access to primary care [1].

### Participants

Males and females aged 40–64 living in eligible low income areas. Only one person per household will be eligible. Participants will be recruited using community-based strategies specific to the individual clusters, devised using participatory research methods [17]. Informed consent will be sought from individual participants.

### Design

Cluster randomized trial, randomly assigning clusters to the BETTER HEALTH: Durham intervention immediately, or to a six-month ‘wait-list control’ prior to the intervention (Table 1).

**Table 1:** SPIRIT flow diagram of BETTER HEALTH: Durham Cluster Randomized Trial

Timepoint	*-t*_*1*_	*t*_*0*_	*t*_*1*_ *(t*_*1*_ *= t*_*0*_ *+ 6 months)*
Cluster randomization	X		
Individual enrollment			
Eligibility screen		X	
Informed consent		X	
Baseline assessments			
BETTER Health Survey (First Visit) [8]		X	
Determination of CDPS eligibility (Table 2)			
MOS Social Supports Scale [20]		X	
Income Security Survey [21]		X	
Food Security Survey [22]		X	
Intervention			
Prevention meeting			
Immediate intervention clusters		X	
Wait-list control clusters			X
Outcome assessment			
BETTER Health Survey (Followup Visit) [8]			
Immediate intervention clusters			X
Wait-list control clusters			X prior to prevention meeting

### Sampling frame and randomization

The sampling frame consists of 16 clusters, which are aggregations of census dissemination areas, identified using the methods described in our prior work [1]. All clusters are categorized in the lowest quintile of urban median household income. The average cluster size is 342 persons in the target age range 40 to 64 years. We will randomly sample 10 clusters to be randomly allocated to immediate intervention or six month wait list control. Random allocation of clusters will be conducted using a random number generator function in the statistical package R, conducted at the level of the cluster using simple random sampling. The complete statistical code will be written in R by the co-principal investigator for biostatistics and implemented simultaneously so that no allocation concealment is necessary.

### Adaptation of BETTER

We will use community-based participatory research (CBPR) principles to guide the adaptation of BETTER. CBPR is a “collaborative research approach that is designed to ensure and establish structures for participation by communities affected by the issue being studied, in all aspects of the research process to improve health and well-being through taking action including social change” [17]. A CBPR approach has improved the quality of interventions in a variety of community settings [17, 18]. We have convened a Community Advisory Committee consisting of a range of stakeholders and residents of low income areas to provide advice on the adaptation of BETTER, recruitment strategies and community engagement throughout the study.

The specific process of adaption of BETTER will follow the ADAPT-ITT model [19]. Stakeholders including prevention practitioners, nurse practitioners, public health professionals, primary care providers, and community stakeholders will be invited to a meeting to learn about BETTER HEALTH: Durham and overall study goals and approach. Subsequently, research team members will conduct small group meetings with members of the public who are potentially eligible for BETTER HEALTH: Durham, to discuss specific needs, and the fit of BETTER HEALTH: Durham to those needs. Extensive notes will be taken at the meetings to document all suggestions. Adaptations of BETTER HEALTH: Durham will be made considering the needs of the community, balanced with fidelity to core elements of BETTER, and discussed in follow up meetings.

### Primary care engagement strategy

Some participants in BETTER HEALTH: Durham will not have a regular PCP, and others who have a regular PCP may have difficulty accessing their PCP. A primary care engagement strategy will identify PCPs willing to accept participants lacking a PCP who wish to have one.

### Data collection and determination of CDPS eligibility

The entry survey will be a modification of the BETTER Health Survey (First Visit) [8], and will be administered to consenting participants as an interview by a research assistant, rather than self-administered, as in the first BETTER trial, because of anticipated low levels of functional literacy. The categories of self-reported income to be collected by the survey will be reset to reflect the focus of low income areas. The survey will include components of the RAND Medical Outcomes Study Social Support Scale items [20], and items pertaining to income security [21] and food security [22]. Responses to these items may inform the approach of the prevention practitioner to the individual participant, and may be associated with the participants’ choices of CDPS goals and their accomplishment.

Eligibility for CDPS actions will be determined exclusively by self-report of previous and current participation in CDPS actions in the survey, unlike in the first BETTER trial, in which eligibility, previous, and current participation were determined from EMRs and the BETTER Health Survey (First Visit) [8]. The maximum number of eligible CDPS actions will be reduced from twenty-eight in the first BETTER trial to seventeen in ‘BETTER HEALTH: Durham’ because of the difference in data sources for determination of eligible CDPS, as demonstrated in Table 2. Those CDPS actions excluded from the first BETTER trial required information from the EMR, for which there is no equivalent self-report.

**Table 2:** Comparison of Chronic Disease Prevention and Screening actions eligible for the composite outcome measure in the first BETTER trial to BETTER HEALTH: Durham

	Better	Better Health: Durham
1	Fasting blood sugar screening	yes
2	Fasting blood sugar monitoring	no
3	Blood pressure screening	yes
4	Blood pressure monitoring	yes
5	Hypertension treatment	no
6	Framingham calculated	Changed to LDL measurement
7	Framingham improved	no
8	LDL improved	no
9	Cholesterol treatment	no
10	Breast cancer screening	yes
11	Colorectal cancer screening	yes
12	Cervical cancer screening	yes
13	BMI screening	yes
14	Waist circumference measured	yes
15	Weight control	yes
16	Weight control referral	yes
17	Smoking screening	no
18	Smoking cessation	yes
19	Smoking cessation referral	yes
20	Alcohol screening	no
21	Alcohol control	yes
22	Alcohol control referral	yes
23	Physical activity screening	no
24	Physical activity > = 90 min / week	yes
25	Physical activity program referral	yes
26	Nutrition screening	no
27	Healthy diet score improved	yes
28	Nutrition counselling referral	yes

Survey data will be entered electronically directly to a database application particularly created for BETTER HEALTH: Durham using the REDCAP platform [23] and housed on a secure server the Applied Health Research Centre, St Michael’s Hospital, Toronto. The database application will automatically determine the eligible CDPS actions for each participant based on an adaptation of the BETTER Algorithm [8] and generate an adapted and populated version of the BETTER Prevention Prescription (a list of eligible CDPS actions and recommended periodicity) [8].

### Intervention and outcome assessment

At the completion of the baseline survey, all participants in all clusters will be offered educational materials available to the public from the Durham Region Health Department promoting cancer screening, safe alcohol use, tobacco cessation, weight control, healthy eating and physical activity. Participants residing in the immediate intervention clusters will be offered the adapted BETTER intervention immediately after the baseline survey. Participants who choose screening tests as short-term goals will go to their PCPs to have these done, if they have a PCP; for those who do not have a PCP and cannot be linked promptly to one, the prevention practitioner will arrange to have the chosen screening tests facilitated by a nurse practitioner engaged by the study for this purpose.

Six months after the baseline survey interview, the research assistant will re-administer an outcome survey based on a modification of the BETTER Health Survey (Followup Visit) [8] to participants in both intervention and ‘wait list’ control clusters, and enter the responses online. The database application will compute the number of CDPS actions completed for each participant automatically after entry of the six-month data. Participants residing in ‘wait-list’ control clusters will be offered the adapted BETTER intervention immediately after completion of the outcome survey.

Differences in the collection of information and flow of participants between the first BETTER trial and BETTER HEALTH: Durham are summarized in Table 3.

**Table 3:** Comparison of the first ‘BETTER’ trial to ‘BETTER HEALTH: Durham’

	Better (*in primary care practices)*	Better Health: Durham *(community / public health setting)*
Physical location of prevention practitioner (PP)	In Family Health Team clinics	Imbedded in Durham Region Health Department, for community outreach
Identification of participants	From electronic medical record (EMR)	Community-based recruitment strategies in low income areas
Informed consent	For collection of personal health information, by prevention practitioner	For collection of self-reported personal health information, by research assistant
Identification of completed and current behaviours and activities	Abstraction from EMR, and from self-report in self-administered survey	From self-report responses to survey administered by research assistant, in the community
Survey data collection	Paper, by patient	Electronic, self-report responses to survey administered by research assistant, in the community
Identification of risk factors	Lab tests, survey, EMR	From self-report, as above
Identification of eligible CDPS actions	Prevention practitioner manually extracted and compiled	Electronically identified and compiled from self-report, as above
Prevention meeting and goal-setting by participants	By prevention practitioner in primary care team clinics	By prevention practitioners at various community locations
Strategy to find primary care provider for participants who lack provider.	Not applicable	Prevention practitioners supported by primary care strategy engaging primary care providers near the participant.
Height, weight, waist circumference, blood pressure	EMR entry or by prevention practitioner or primary care provider	By prevention practitioner or primary care provider, self-report on baseline or 6 month survey
Specimen collection for laboratory-based screening	In laboratories by requisition from primary care providers	In laboratories by requisition from primary care providers or from nurse practitioner for participants without primary care provider
Facilitation of goal achievement	Clinic staff, prevention practitioner, links, and self	Prevention practitioners, links, and self
Followup of abnormal results	By primary care physician	By primary care providers, or by nurse practitioners engaged by study if prevention practitioners unable to link participant with primary care provider
Ascertainment of outcomes	Abstraction from EMR and self-report responses at repeat self-administered survey by prevention practitioner	Self-report responses to 6-month survey administered by research assistant
Primary outcome measure	“composite index, expressed as the ratio (multiplied by 100) of the number of eligible CDPS (chronic disease prevention and screening) actions at baseline (denominator) that were subsequently met at follow-up (numerator), measured at the patient level.” (Grunfeld 2013)	

### Qualitative evaluation and analysis plan

We have adapted the approach taken for the evaluation of BETTER 2 in Newfoundland to the evaluation of the adaptation of BETTER [12]. The key questions to guide the qualitative evaluation are: 1. How was BETTER adapted? 2. What has been the impact of BETTER HEALTH: Durham as perceived by stakeholders? 3. What barriers and enablers of BETTER HEALTH: Durham have been encountered? 4. What are the benefits and disadvantages of BETTER HEALTH: Durham?, and 5) How can the implementation of BETTER HEALTH: Durham be sustained?

**Fig. 1:**
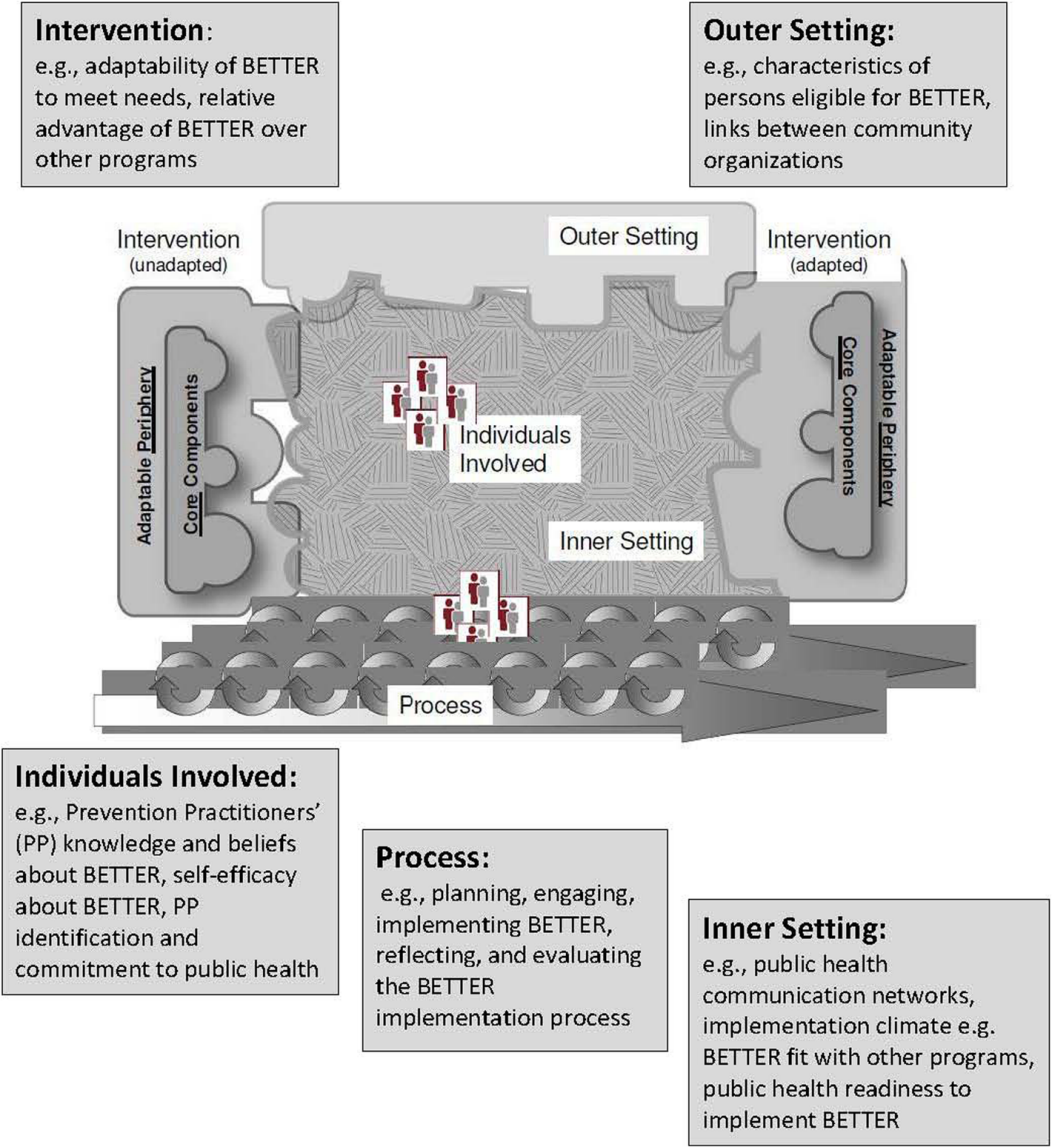
Consolidated Framework for Implementation Research adapted to BETTER HEALTH: Durham. Adapted from: Damschroder [25]

We will use a qualitative approach based on grounded theory [24] and the Consolidated Framework for Implementation Research (CFIR) [25] to evaluate the adaptation and implementation of BETTER (Fig. 1). Grounded theory is a well-known qualitative method suited to examining processes, such as the adaptation of BETTER and the implementation of BETTER HEALTH: Durham in the context of a public health unit in low income areas. CFIR was developed from existing implementation frameworks and illustrates interrelationships among five different domains (Fig. 1). Given the complexity of the adaptation and implementation processes for BETTER, all potentially relevant domains will be considered. Constructs for each domain of CFIR have been described which will facilitate interpretation during data coding and analytic processes [25] .

We will collect qualitative data three times during the project: 1) at the beginning of the study to assist with adaptation and start-up issues (Year 1); 2) after the intervention is established to understand perceived enablers and barriers, benefits and disadvantages (Years 2–3), and 3) near the end of the study (Year 4) to explore sustainability and impact. Data collection strategies will include focus groups and one-to-one, semi-structured interviews with a range of participants: 1) members of the public eligible for BETTER who live in low income clusters that will not be participating as well as clusters that will be participating; 2) prevention practitioners who are providing the BETTER intervention; 3) Medical Officer of Health (MOH) and public health unit; 4) primary care providers; and 5) other community stakeholders. Interview guides will be based on the study objectives and revised periodically to seek contrasting and supporting data. Interviews will be recorded and transcribed verbatim and field notes will be created to document non-verbal and contextual information. The number of interviews or focus groups depends on data saturation. Saturation occurs when data categories are dense and no new or relevant data are being observed [26].

Consistent with grounded theory, we will use the constant comparative method for data analysis. Initially, two team members will code approximately 2–3 transcripts in each data collection phases using an editing style of coding [27]. From the codes identified during this process, a preliminary coding guide will be developed and reviewed with all team members. Subsequently, a research assistant will code the remaining transcripts using the coding guide. We will hold periodic analysis meetings with several team members to review the codes, sort codes into categories and identify main themes [25, 28]. Furthermore, team members will create memos that will document emerging relationships among the codes and categories. We will use data management and analysis software (NVivo 10, QSR International). An audit trail including interview summaries and memos will improve trustworthiness through transparent documentation of all major decisions made during data collection and analysis [29]. Involvement of several members of the research team during the analytic process will ensure that identified themes are consistent with coded data. We will triangulate the data from several sources to provide a full description of the themes.

### Quantitative analysis plan

The main outcome is a composite index score, consisting of the ratio (multiplied by 100) of the number of eligible CDPS actions at baseline (denominator) that have been accomplished at follow-up (numerator) according to self-report, calculated at the level of the individual participant [5]. This index is modelled on the Summary Quality Index (SQUID) method, developed by Nietert in order to evaluate the quality of interventions administered in primary care settings [11]. At study enrollment, the eligibility of each participant for CDPS actions will be determined according to health history and health status. Six months later, the number of ‘eligible’ CDPS actions which are met by each participant will be determined [5]. Analysis of the primary outcome (absolute percent increase in eligible CDPS actions completed or engaged in) will account for the correlation among outcomes which may arise from individuals within the same cluster, by implementing a two-level hierarchical regression model [30]. Specifically, a generalized linear random effects regression model will be constructed in which a cluster-specific random effect, arising from a normal distribution, will be included to account for the dependency among outcomes of individuals within the same cluster [31–34]. The main binary exposure in the hierarchical model will be immediate BETTER compared to ‘wait-list’ control clusters; furthermore any characteristics that were not balanced (between the immediate BETTER and ‘wait-list controls’) from the randomization process will be adjusted for in the hierarchical regression model. In addition to obtaining estimates of the regression parameters, the hierarchical regression model will allow us to investigate the percent of residual or unexplained variation attributable to each level of the hierarchy.

### Sample size calculation

With an equal number of participants in five intervention clusters and five wait-list control clusters (the average number of eligible residents per cluster being 342), a total sample of 120 participants (on average, 12 participants per cluster) will be required to detect an increase of 30% or greater in the composite index score (reflecting additional actions met among the intervention clusters (compared to the control clusters), as observed in the first BETTER cluster randomized trial [5]. The calculation is based on a two-sample comparison of means with 80% power and alpha = 0.05. The calculation accounts for the design effect (correction factor determined as (1 + (m-1)*rho)) arising from the clustered design, with intracluster correlation coefficient = 0.237, as in the original BETTER cluster randomized trial [5]. The ICC is a measure of the relatedness of the clustered data [31].

### Knowledge translation (KT) plans

**Fig. 2:**
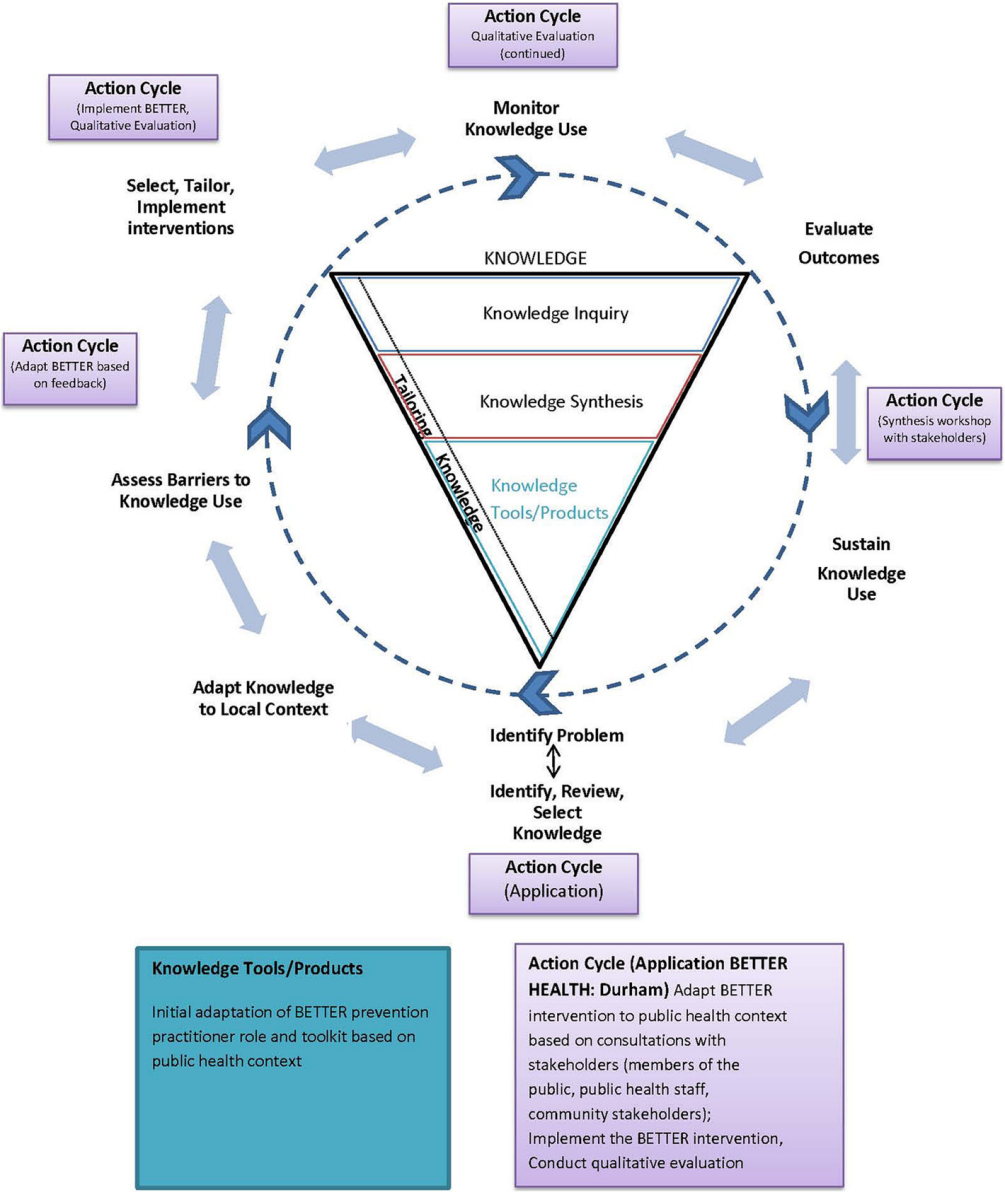
Adapted Knowledge to Action Process for BETTER HEALTH: Durham. Adapted from Canadian Institues for Health Research [35]

Integrated and end-of-grant KT activities are central to the adaption and implementation of the BETTER intervention in a community context [16]. We will use the Knowledge to Action process [35] as a guiding framework (Fig. 2). Key stakeholders including the BETTER HEALTH: Durham Community Advisory Committee will be involved in the development of comprehensive integrated and end-of-grant KT plan.

#### Integrated KT

KT will be ongoing throughout all phases of the study including adaptation of BETTER, implementation and evaluation. The CBPR approach used in this study will facilitate integrated KT. For example, staff members of the Durham Region Health Department have been closely involved in the creation of the proposal. During the adaptation of BETTER, we will consult with key stakeholders including members of the public who are eligible for CDPS activities in eligible clusters, and leaders of community organizations who have insights of different facets of the adaptation and implementation process, and who will have a stake in the study results. We anticipate that bidirectional knowledge exchange will occur in one to one, small and large group interactive face-to-face meetings. Project updates will be provided via social media and websites and will be presented at public health knowledge exchange fora. Early communication with stakeholders will shape subsequent messages and avenues for integrated KT. The effectiveness of integrated KT will be assessed as part of the qualitative evaluation. Early evaluation will uncover strengths and weaknesses of the integrated KT approach so that new strategies can be developed if needed.

#### End-of-grant KT

We will hold a summative KT workshop at the end of the study involving all stakeholders. In addition, multiple small group meetings will be held in the community to reach all stakeholders including residents of eligible clusters. Study briefing notes will be created and tailored to different stakeholder needs (determined using data from the qualitative evaluation). Investigators on this study will facilitate communication of study results to key knowledge users in public health, provincial government including health and cancer control, and primary care via webinars or other means tailored to the specific audience. Traditional end-of-grant activities will include abstracts for academic presentations and manuscripts submitted to peer-reviewed open access journals.

## Discussion

In Ontario, CDPS are mandates of primary care and also public health sector. BETTER HEALTH: Durham will adapt a successful strategy from the primary care setting to the public health setting, for a population simultaneously at elevated risk for chronic disease morbidity, and less likely to be engaged in CDPS activities and primary care, compared to the overall population. In addition to providing important information about the effectiveness of the adapted BETTER for this population in the public health setting, BETTER HEALTH: Durham will make important observations about the implementation process of this intervention as well as the collaboration between the public health and the primary care sectors.

## Abbreviations

BETTER: Building on Existing Tools To Improve Chronic Disease Prevention and Screening
CBPR: Community-Based Participatory Research
CCSRI: Canadian Cancer Society Research Institute
CDPS: Chronic Disease Prevention and Screening
CFIR: Consolidated Framework for Implementation Research
CIHR: Canadian Institutes for Health Research
EMR: Electronic Medical Record
ICC: Intra-class correlation coefficient
KT: Knowledge Translation
MOH: Medical Officer of Health
PCP: Primary Care Provider
SQUID: Summary Quality Index
